# Design and Study of Pulsed Eddy Current Sensor for Detecting Surface Defects in Small-Diameter Bars

**DOI:** 10.3390/s24248063

**Published:** 2024-12-18

**Authors:** Lei Han, Yi Jiang, Ming Yuan

**Affiliations:** 1 School of Electrical Engineering, Nanjing Vocational University of Industry Technology, Nanjing 210023, China; 2021101294@niit.edu.cn; 2 College of Automation & College of Artificial Intelligence, Nanjing University of Posts and Telecommunications, Nanjing 210023, China; yuanming@njupt.edu.cn

**Keywords:** impulse eddy current testing, small-diameter bars, non-destructive testing, eddy current disturbance, quantitative determination

## Abstract

The design and study of pulsed eddy current sensors for detecting surface defects in small-diameter rods are highly significant. Accurate detection and identification of surface defects in small-diameter rods may be attained by the ongoing optimization of sensor design and enhancement of detection technologies. This article presents the construction of a non-coaxial differential eddy current sensor (Tx-Rx sensor) and examines the detection of surface defects in a small diameter bar. A COMSOL 3D model is developed to examine the variations in eddy current distribution and defect signal characteristics between the plate and rod components. The position of the excitation coil on the bar and the eddy current disruption around the defect are examined. Additionally, a Tx-Rx sensor has been developed and enhanced concerning coil dimensions, coil separation, and elevation height. An experimental system is established to detect bar structures with surface defects of varying depths, and a model correlating differential signal attenuation with defect depth is proposed, achieving a quantitative relative error of less than 5%, thereby offering a reference for the quantitative detection of bar surface defects.

## 1. Introduction

Metal bars are extensively utilized as a crucial structural component in aerospace, military, energy, and several other industries [1]. These structural materials are often affected by surface defects, including friction and corrosion, during usage. If not identified and eliminated promptly, the reliability of critical equipment in the aerospace and military sectors is severely compromised, leading to component failures that result in casualties and substantial economic losses [2]. A non-destructive testing investigation of the surface defects of metal bars is necessary.

Presently, four detection technologies are commonly utilized to identify metallic materials: ultrasonic detection [3,4], X-ray detection [5], laser ultrasonic detection [6,7], and eddy current detection [8,9]. Ultrasonic detection necessitates a coupling agent to link the sensor to the metal surface; however, the prevalence of thick oxide coatings on many metal surfaces limits this method [10,11]. The detection environment requirements are stringent due to the radiation source, which may cause pollution concerns during actual field detection processes. Non-contact detection via laser ultrasonic technology is feasible; however, the efficacy of interferometer focusing is compromised by surface roughness and the properties of metal surfaces, resulting in diminished detection performance, and the apparatus is expensive [12]. Pulsed eddy current testing (PEC), due to its characteristics such as extensive range, significant detection depth, and straightforward quantification, serves a crucial function in the assessment and evaluation of metal materials. It is a pivotal non-destructive testing technology that ensures the integrity of metals during manufacturing and usage [13,14,15,16,17].

Le et al. [18] utilized a finite element model to assess the distribution of eddy currents in pipeline problems within the context of theoretical eddy current testing research. Xing Lingling [19] examined the temporal distribution characteristics of transient eddy current fields on a uniformly thick planar conductor with thin cracks, utilizing the finite-boundary element coupling method. G. Y. Tian et al. [20,21] introduced a novel feature known as the time rise point, derived from the differential analysis of detection signals with and without defects, thereby acquiring defect information by converting the time rise point of differential signals influenced by defects into an eddy current field. Zhou Deqiang et al. [22] analyzed the spectrum amplitude of the pulsed eddy current differential signal post-Fourier transform and determined that the fundamental frequency and amplitude of higher harmonic components of the defect detection signal exhibit a linear correlation with the defect’s depth. Gabriel et al. [23] initially developed a pulsed eddy current defect detection model for the quantitative assessment of defects and acquired detection signals. G. Preda et al. [24] created a neural network-based mapping model, established the correlation between detection signals and defect contours, and executed two-dimensional defect reconstruction on thicker plates. Wang Li et al. [25] employed the skin effect to delineate the contours of deep corrosion defects by analyzing the harmonic signals of pulsed eddy current detection at various frequencies. Pulsed eddy current testing, especially with conventional coaxial sensors, has been extensively studied in the context of large-diameter metal pipes and thick plates [26]. However, the signal in the receiving coil is significantly influenced by the magnetic field produced by the excitation coil, leading to a comparatively poor signal-to-noise ratio for this probe type [27]. Nevertheless, there exists a paucity of studies regarding eddy current distribution, the distinguishing characteristics of defect signals, and the quantitative detection of surface defects on small-diameter bars that require additional investigation.

The current sensor design is essential for detecting small diameter bars with pulsed eddy current detection technology. Volumetric sensors, including Bobbin sensors [28,29] and pancake sensors [30], are frequently employed in eddy current sensors. Despite the rapid detection speed of the Bobbin sensor, its detection capabilities are limited [31,32]. Pancake sensors have superior detection sensitivity; nonetheless, they possess notable disadvantages, including slow detection speed, restricted sensor lifespan, and significant susceptibility to lift effects [33,34]. Moreover, the flexible sensor conforms effectively to the curve of the tiny bar’s surface; however, it possesses a complex geometric configuration, an intricate operational mode, and incurs substantial costs [35,36,37,38]. Additionally, the excessive pulse energy is detrimental to film integrity, hindering detection. The differential eddy current probe has the benefits of mitigating common-mode signals and exhibiting reduced sensitivity to lift-off, thereby necessitating the design and implementation of the differential eddy current sensor for the examination of tiny diameter bars.

Consequently, to investigate the eddy current distribution and defect signal characteristics of the Tx-Rx sensor on small-diameter rod components, this paper initially employs COMSOL 3D model simulation to examine the disparities in eddy current distribution and defect signal characteristics between Tx-Rx sensor plates and rod components, followed by a comparison of the current density distribution of plates and rod components at varying frequencies. Analyzed the placement of the excitation coil on the rod and the eddy current interference surrounding the defect. Subsequently, the Tx-Rx sensor was produced, and its calibration was performed according to the dimensions of the coil, the spacing between coils, and the lift-off distance. An experimental system was created to recognize rod structures with surface defects of differing depths, and a model correlating differential signal attenuation with defect depth was produced, enabling quantitative defect identification. This work establishes a research basis and introduces novel techniques for identifying surface defects in small-diameter rods.

## 2. Principle of Pulsed Eddy Current Testing Based on Tx-Rx Sensor

**Figure 1:**
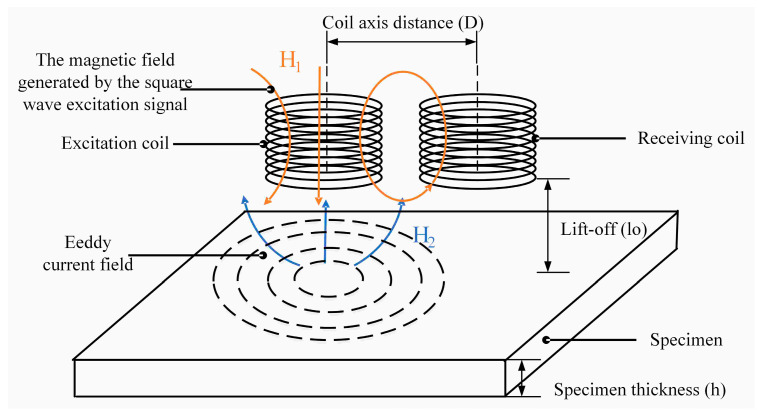
Pulsed eddy current Tx-Rx sensor detection schematic.

The pulsed eddy current sensor is a mutual inductance coil that consists of an exciting coil and a receiving coil. A schematic diagram of the pulsed eddy current detection principle based on the non-coaxial pulsed eddy current sensor is shown in Figure 1. As the excitation signal, square waves with a certain duty cycle travel through the excitation coil. When the square wave signal is on the rising or falling edge, the coil generates a changing magnetic field H_1_, and due to the change in magnetic flux, an induced electromotive force is created in both the excitation and receiving coils [39,40]. Self-inductance is defined as the ratio of the induced electromotive force in the excitation coil to the current in the excitation coil. The mutual inductance between the coils is defined as the ratio of the induced electromotive force in the receiving coil to the stimulating current. The magnetic field created by the square wave signal in the excitation coil is attached to the test specimen at the same time, and an eddy current is induced in the test specimen. According to Lenz’s law, the eddy current in the specimen creates a magnetic field H_2_ opposite to the exciting magnetic field, and this component of the magnetic field induces the electromotive force in both coils at the same time, modifying their mutual inductance. The signal in the receiving coil is the sum of the induced electromotive force created by the excitation magnetic field H_1_ in the receiving coil and the induced electromotive force generated by the specimen eddy current field H_2_ in the receiving coil. The signal connected to the receiving coil through the specimen’s eddy current field H_2_ provides information such as the thickness and defect of the portion to be examined. Differential processing is frequently conducted on the signal in the detecting coil to obtain the differential signal in order to retrieve this information. In general, the differential signal is derived by subtracting the reference signal from the standard specimen and the detection signal from the test piece. The signal in the receiving coil is determined by the coupling between the specimen, the excitation coil, and the receiving coil, and the coupling of the three is directly connected to coil spacing, lift distance, and specimen condition.

## 3. Finite Element Simulation

### 3.1. Simulation Model

**Figure 2:**
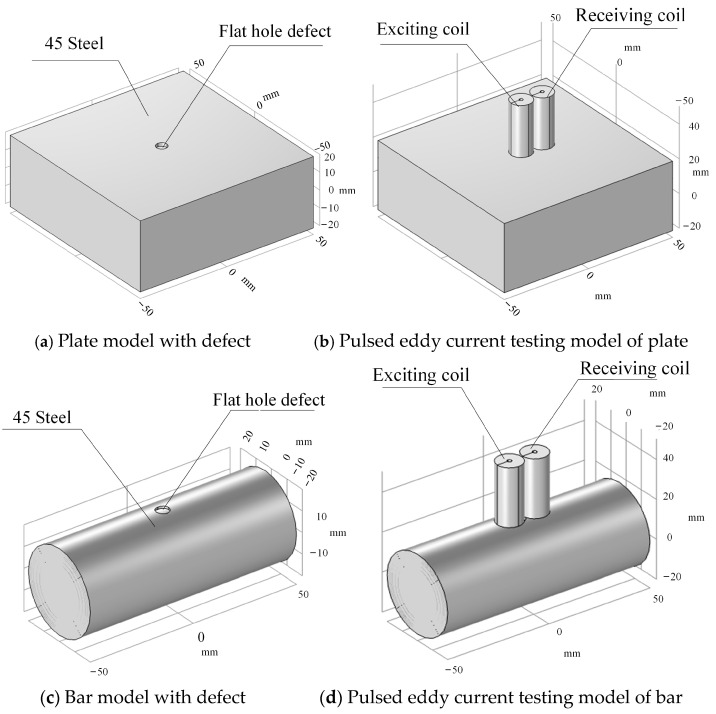
Schematic diagram of pulsed eddy current testing model.

To examine the electromagnetic field distribution of a tiny bar specimen, two model sets are created utilizing the material parameters specified in Table 1. Figure 2a,c depicts two specimens: one is a plate specimen measuring 40 mm in thickness, 100 mm in length, and 100 mm in breadth, while the other is a tiny bar specimen with an outer diameter of 40 mm and a length of 100 mm. The two models analyze the variation trend of the surface eddy currents in members of differing geometries, providing a reference for recognizing the curved surfaces of the bar. Additionally, the device utilizes a Tx-Rx sensor, including a one-point-one receiver. Table 2 presents the specific parameters, and Figure 2b,d illustrate the model.

The grid element is constructed on a physical field control grid and divided by a tetrahedral grid. The advantage of the sensor design compared to the coaxial sensor is its capacity to mitigate the significant impact caused by slight lift-off and improve the overall stability of the detection system. The mesh information statistics in COMSOL indicate that the surface mesh of the plate model has 28,860 complete vertices, 435,696 tetrahedral elements, and an average cell mesh mass of 0.9194. The surface mesh of the rod-like model generated 8024 full vertices and 23,998 tetrahedral meshes, and the average cell mesh mass was 0.9281. Moreover, the coil excitation magnetic field in space constitutes an open domain challenge, necessitating the preservation of a designated air region surrounding the detecting environment to prevent enforced truncation and provide adequate attenuation, hence eliminating any forced truncation.

The examination of simulation coil geometry and the transient solver are configured in the model-solving section. Coil geometry analysis is an essential procedure for models using coils. Pulsed eddy current detection is a transitory method that primarily examines signals at the rising and falling edges of a square wave. The analysis focuses on the properties of the 10 Hz square wave signal with a 50% duty cycle, namely before and after 0 s and 0.05 s. In phase 2 of the study, pick the transient model, configure the unit to seconds in the research settings, and set the solution time step to 10^−5^ s.

A flat hole defect measuring 5 mm in diameter and 2 mm in depth is fabricated on two models to simulate wear and pitting defects on the material surface, facilitating the assessment of surface degradation’s effect on the pulsed eddy current detection signal. The simulation investigates the gradient fluctuation of the defect depth in the flat bottom hole on the bar to further analyze the relationship between defect depth and detection signal, specifically at depths of 2 mm, 4 mm, and 6 mm. The detection outcomes for different depth defects are obtained by adjusting the parameters of the defect depth in the model, facilitating the execution of pulse analysis.

### 3.2. Analysis of Electromagnetic Field Distribution Simulation Results

**Figure 3:**
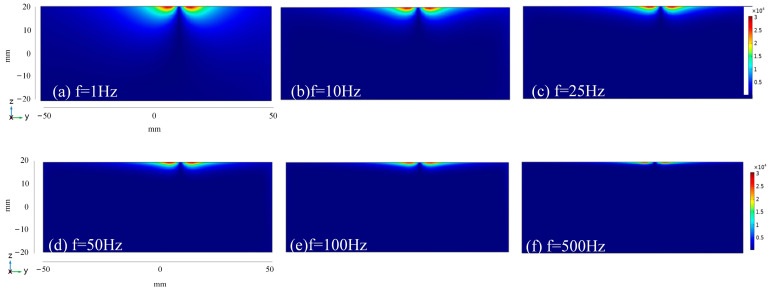
Current density simulation cloud image of plate cross-section at different frequencies.

**Figure 4:**
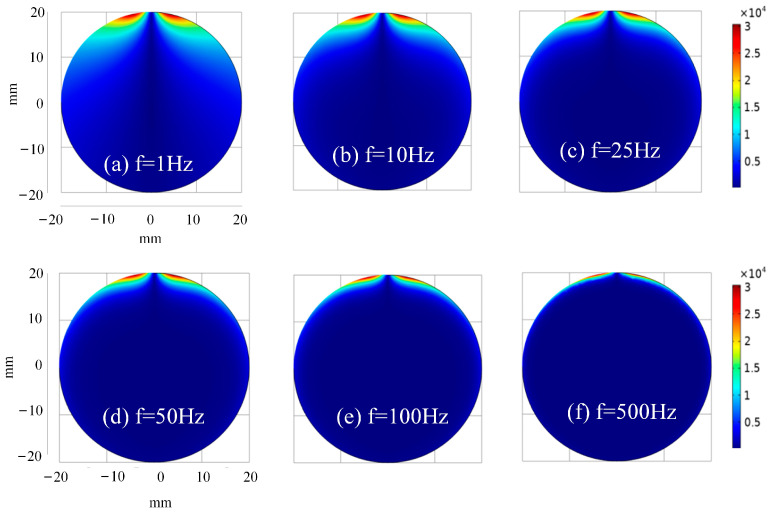
Current density simulation cloud image of bar cross-section at different frequencies.

To investigate the current density distribution of specimens under various frequency stimulation, the simulation set the falling edge duration to 1 ms and chose square waves with frequencies of 1 Hz, 10 Hz, 25 Hz, 50 Hz, 100 Hz, and 500 Hz, and a duty cycle of 50% as excitation sources for investigation. The section of the specimen directly below the excitation coil is selected for analysis. The current density cloud diagram of the plate is shown in Figure 3, and that of the small bar is shown in Figure 4.

Comparing Figure 3 with Figure 4 reveals that, at identical frequencies, the material shape does not influence the variation range of the overall current density mode value, whereas it does affect the relative penetration depth. In the plate seen in Figure 3a, the eddy current initially diffuses outward over the upper surface, centered on the axis of the excitation coil, and progressively penetrates below. Due to the curvature of the bar’s surface, as seen in Figure 4a, the eddy current disperses outward along the surface, resembling diffusion in the direction of increasing specimen depth. Moreover, the eddy current would penetrate vertically and be superimposed at a certain depth of the specimen, resulting in a higher current density modulus value at the corresponding location of the bar compared to that of the sheet at the same depth. Consequently, in comparison to the plate material, the bar subjected to the same frequency stimulation will exhibit a greater eddy penetration depth due to its unique spatial configuration.

**Figure 5:**
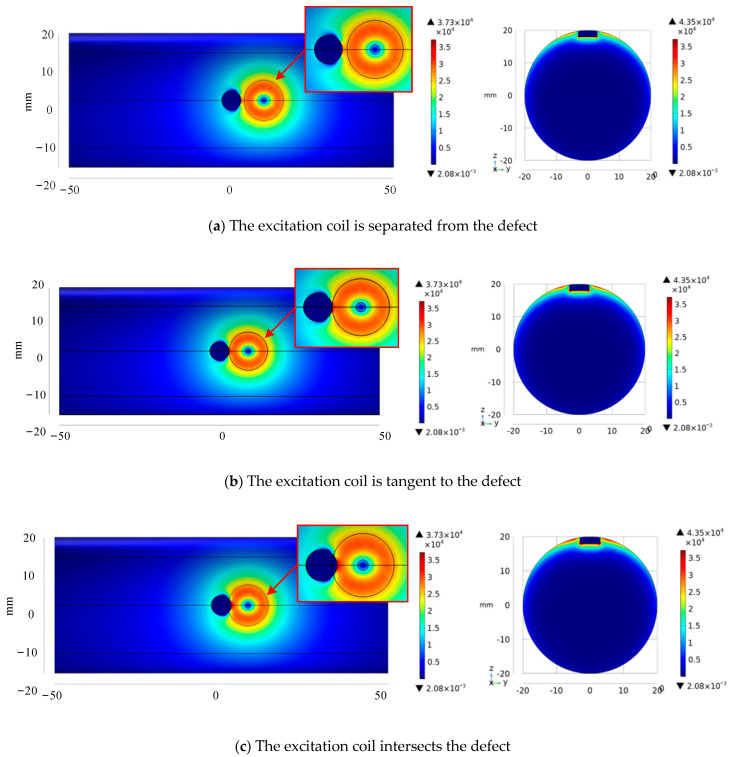
Simulation cloud image of eddy current disturbance at defect under different excitation coil positions.

To highlight the disturbance of eddy current distribution in the specimen during defect detection, three types of excitation coil positions are designed based on the simulation of the bar specimen, which is separated from the defect, tangential, and intersecting. Figure 5 depicts the cloud picture findings. As the excitation coil approaches the defect, the current density distribution underneath it will circumvent the defect as a whole, and a region with a higher current density mode will appear at the defect border, according to the current density distribution on the surface of the specimen. The section diagram of the specimen shows that as the exciting coil approaches the defect, the current density modulus value on the corresponding section gradually increases; that is, when the exciting coil intersects with the defect, the detection coil is placed in the surrounding area, which is conducive to capturing the defect’s disturbance on the eddy current field. As a result, the excitation coil location depicted in Figure 5c has the best relative effect, and this set of simulations serves as a guideline for coil placement position in the analysis of time-domain defect response signals.

### 3.3. Results and Analysis of Defect Response Signal

**Figure 6:**
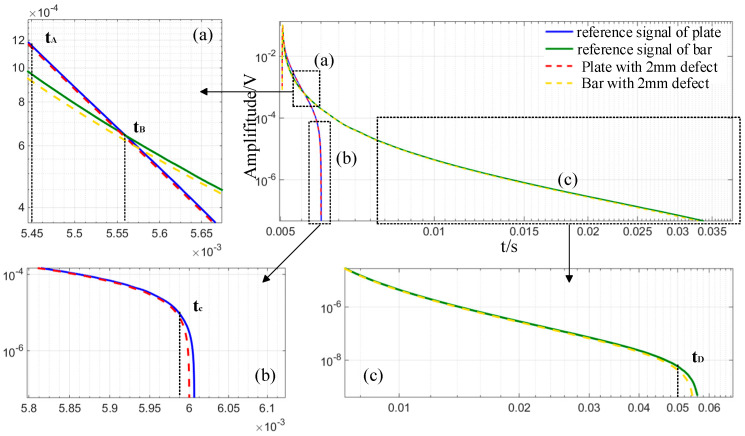
Time-domain signal simulation results of induced voltage of bar and plate.

Figure 6 illustrates the time-domain induced voltage signals of plates and bars, both with and without faults, derived from the double-logarithmic coordinate system. Figure 6a–c are enlarged versions of the black dotted boxes in Figure 6. The region preceding the subsequent rising edge is analyzed following the falling edge. The time-domain signal range of the induced voltage from the detecting coil examined in this work is 5 ms to 10 ms at an excitation frequency of 10 Hz. The results in Figure 6 indicate that the observed voltage signals of bars and plates exhibit a temporal decline with markedly different decay rates. Figure 6a illustrates that prior to t_B_, the induced voltage signal of the plate exceeds that of the bar under identical conditions. At t_B_, the measured voltage signals of the two configurations are essentially identical. After t_B_, the induced voltage of the plate decays rapidly, while that of the bar decays slowly. Because the effective volume of the bar in space is more concentrated and the surface area situated in the effective electromagnetic field is bigger, the eddy current is confined by the circumferential border of the bar and will reflect at the top boundary. However, because of the plate’s boundary structure, the reflection will occur after the eddy current has diffused to the lower surface, so the attenuation of the detection signal of the bar before the t_B_ moment is slower than that of the plate and the attenuation speed after the t_B_ moment is lower than that of the plate.

In addition to the specimen’s morphology affecting the voltage signal variation, defects inside the specimen are also directly linked to the voltage signal. Figure 6 illustrates the time-domain signals of induced voltage for the plate and bar, both in the absence of defects and in the presence of holes, represented by dashed lines. Figure 6b,c illustrate that the attenuation rate of the detection signal is consistently greater in the presence of defects compared to the absence of defects for both the plate and the bar. The absence of conductive material in the deficient specimen accelerates the attenuation of the produced current in the associated specimen.

**Figure 7:**
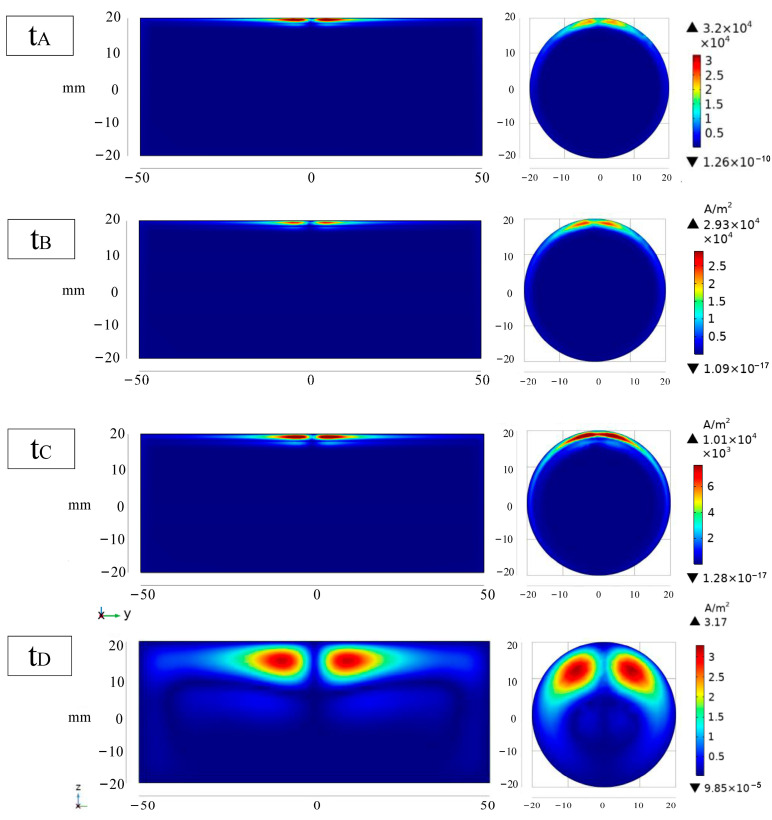
Simulation cloud image of cross-section current density distribution of bar and plate without defect.

To visualize the change in current density in the specimen, the simulation is run with the cross sections of plate and bar under non-destructive conditions, and the current density distribution cloud maps corresponding to t_A_, t_B_, t_C_, and t_D_, which are representative of the four moments in Figure 6, are drawn. The modulus value and fraction of the region with high current density in the plate at t_A_ instant is larger than those of the bar, as illustrated in Figure 7. At t_B_ time, the current density modulus values of the two are identical, and the induced voltage of the bar is greater than that of the plate at t_C_ and t_D_ time, as is the corresponding area with higher current density modulus values.

**Figure 8:**
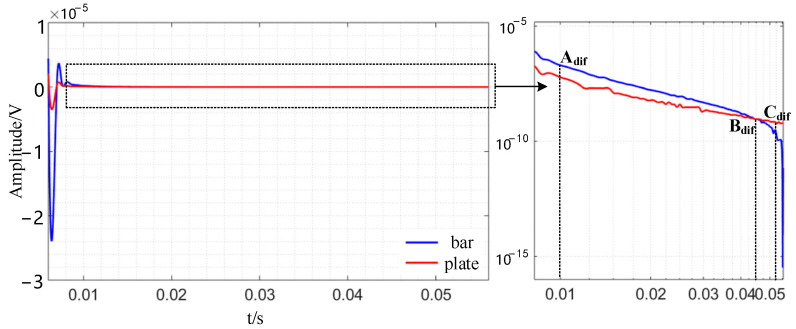
Simulation results of differential time-domain signals of plate and bar.

In addition, differential calculation of the induced voltage signal with and without faults helps better illustrate the time-domain effect of defects. The plate and bar signals are analyzed individually, and Figure 8 shows the results. Under the conventional rectangular coordinate system, the differential signal will change greatly early on. The bar’s peak–peak value is much higher than the plate’s before 0.01s, and its attenuation rate is slower. The magnetic focusing qualities of the ferromagnetic conductor and the high reflection condition of the specimen’s border allow electromagnetic energy to be confined to a smaller volume.

**Figure 9:**
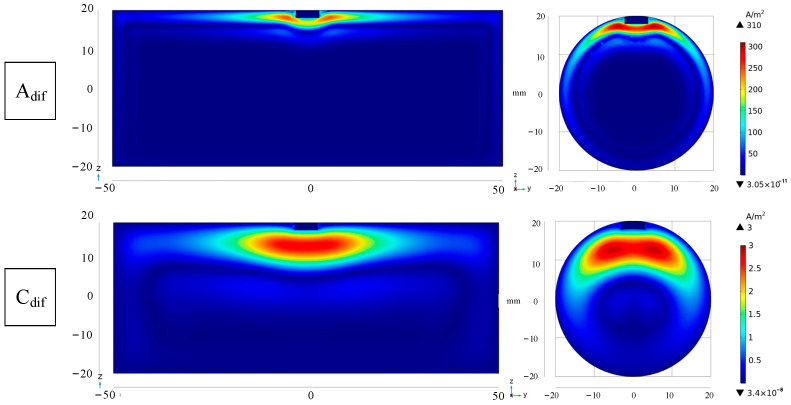
Simulation cloud image of cross-section current density distribution of bar and plate with defects.

The differential signal amplitude of the plate is greater than that of the bar before the Bdif moment, and the differential signal amplitude of the plate is smaller than that of the bar after the B_dif_ moment, as shown in Figure 8, and the attenuation speed of the differential signal of the plate shows an increasing trend. In order to compare the current density distribution of the cross-section at the defect position of the specimen at the corresponding time, the simulation selected the current density cloud image at the A_dif_ time and the Cdif time, as shown in Figure 9. As seen in Figure 9, the emergence of defects will alter the current density distribution inside the specimen. The impact of defects on the eddy current distribution inside the specimen may be easily identified by circling around the bottom of the defects.

**Figure 10:**
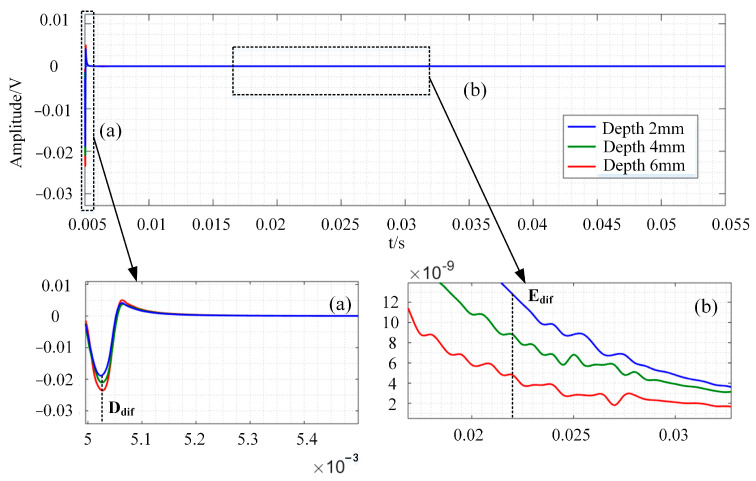
Differential signal simulation results of bar cross-sections with different depth defects.

**Figure 11:**
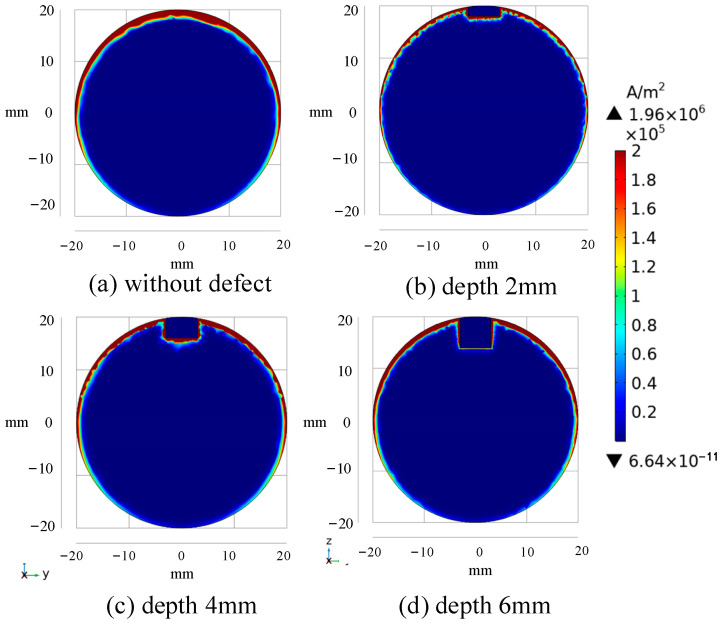
Eddy current distribution simulation cloud image of bar section at D_dif_ time (t = 0.00502 s).

**Figure 12:**
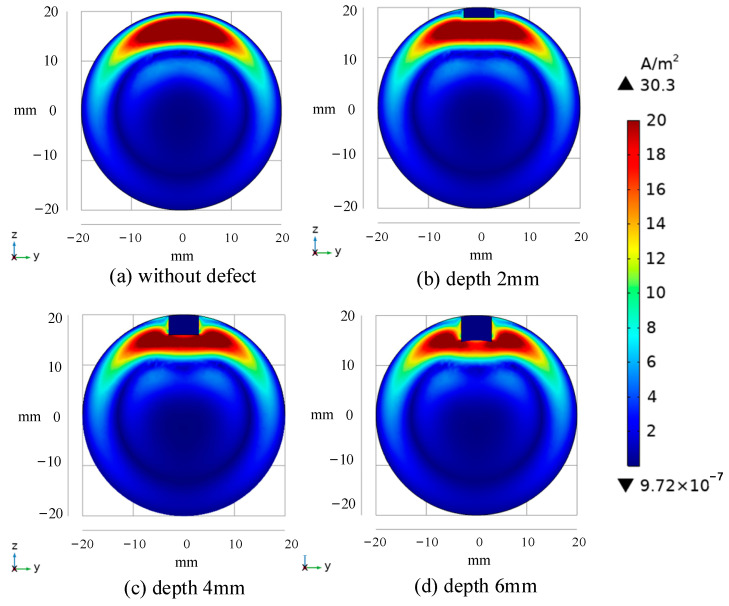
Eddy current distribution simulation cloud image of bar cross-section under different depth defects at E_dif_ time (t = 0.022 s).

The bar models with defects of different depths are further designed, and the differential signals under different defect depths are calculated, with the results shown in Figure 10. Figure 10a,b are enlarged versions of the black dotted boxes in Figure 10. As defects of different depths have different degrees of eddy current disturbance, it can be seen from Figure 10a that the signal peak-to-peak value increases with the increase in defect depth. When combined with Figure 11, the cloud of cross-section current density distribution at the time of D_dif_ (t = 0.00502 s), it is possible to conclude that the ferromagnetic conductor has a magnetic focusing property and that high reflection conditions exist at the specimen’s boundary. As a result, electromagnetic energy is easily accumulated in a smaller volume, resulting in a rise in the eddy current density area in the specimen at this time, resulting in a greater trough absolute value for the associated differential signal at a significant depth defect. Figure 10b depicts the results of the differential signal attenuation in the middle and late phases using a single logarithmic coordinate system. It can be shown that as the defect depth increases, the differential signal has a tiny amplitude and a fast attenuation speed. The eddy current distribution cloud diagram of the section corresponding to E_dif_ (t = 0.022 s) is shown in See Figure 12. The diagram shows that as the defect increases, the current density distribution circles around the bottom of the defect and has a greater density current distribution in the z direction near both sides. The differential signal shows quicker attenuation as the fault widens because the effective specimen volume in the magnetic field reduces, and the component that prevents eddy current attenuation declines. When the defect depth is unknown, the late amplitude of the differential signal, attenuation, and defect depth can be linked to quantify the defect.

## 4. Probe Design

**Figure 13:**
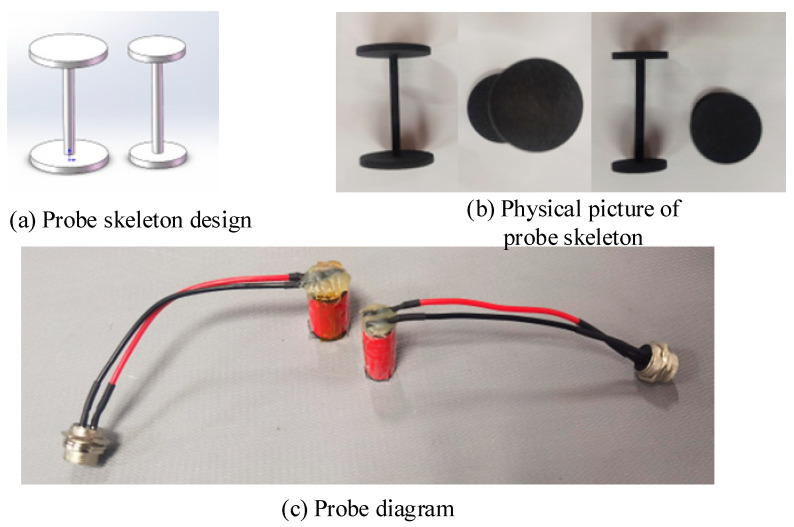
Tx-Rx sensor.

The major research objective of this study is the detection of surface defects on the bar. Considering that the coil’s dimensions, the number of turns, and the wire diameter influence the probe’s coverage area, two coil specifications are developed to evaluate the optimal structural combination of the probe in conjunction with the geometric configuration of the bar and the pulsed eddy current simulation model. The excitation and detection coils are constructed according to the parameters listed in Table 3, utilizing a nylon skeleton produced through 3D printing. The design is illustrated in Figure 13a, while the physical representation is depicted in Figure 13b. The wire employed is copper enameled wire, and the wiring is adapted to an aviation connector to mitigate signal interference. Figure 13c illustrates the finished physical coil.

### 4.1. Signal Influencing by Coil Structure

**Figure 14:**
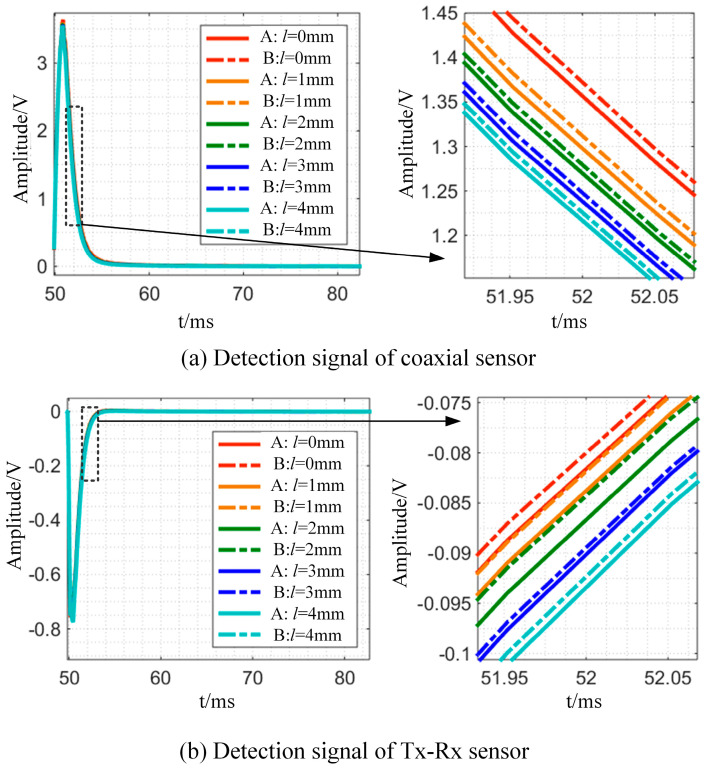
The experimental results of sensors with different structures under varying lift-off.

In order to verify that the Tx-Rx sensor is less affected by lift-off changes, this section compares the detection signals of the Tx-Rx sensor with those of a coaxial probe and conducts experiments on a bar with a diameter of 25 mm (A) and a bar with a diameter of d_2_ = 20 mm (B). The detecting coil of coaxial construction, for example, has an inner diameter of 42 mm, an outer diameter of 60 mm, a height of 22 mm, and a wire diameter of 0.5 mm, with 3600 turns in total. For excitation, a 150 mA square wave current at 10 Hz and 50% duty cycle is used. In the experiment, the lifting height *l* is varied in steps of 0 mm, 1 mm, 2 mm, 3 mm, and 4 mm, and the difference in detected signal changes between the two constructions is examined, as shown in Figure 14.

As demonstrated in Figure 14, lift-off variation affects the detection signal more in the coaxial probe than in the Tx-Rx probe. The coaxial structure detects voltage changes of 0.15 V at t = 52 ms, whereas the Tx-Rx sensor detects 0.01 V. When the coaxial structure sensor is detected, the magnetic field passing through the detection coil is the same as the excitation coil’s magnetic field, while the Tx-Rx sensor’s detection coil is on one side of the excitation coil and has the opposite magnetic field.

The experimental results show that the Tx-Rx sensor is less affected by lift-off modification, improving detection stability and making it suitable for tiny bar detection.

### 4.2. The Influence of Coil Combination on Detection

**Figure 15:**
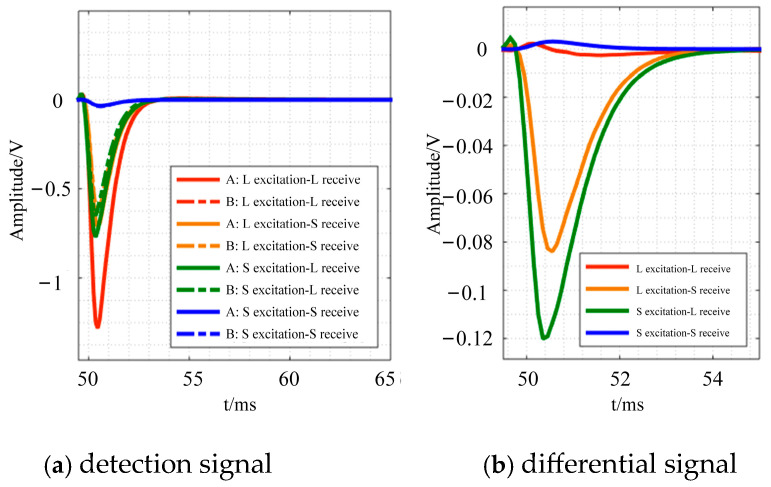
Experimental results of detection signals of different coil combinations.

This section describes how different sensor combinations affect the detection signal. In the experiment, two types of coils from Table 3 are joined in pairs to function as the excitation and receiving coils. The test results are shown in Figure 15. As shown in Figure 15, the detection signal amplitude of the combination mode with a large-sized coil (L) as excitation and a small-sized coil (S) as receiving is large, but when the difference between the A detection signal and the B detection signal is calculated, it is discovered that this combination mode has a poor perception of the thickness thinning of the measured part. In contrast, the combination of a small-sized coil for excitation and a large-sized coil for reception has apparent advantages, and the detection signal of this combination is reasonably excellent, with noticeable difference signal changes. The experimental findings demonstrate that the combination of a small-sized coil for excitation and a large-sized coil for reception is favorable for sensing the signal shift produced by the missing material in defect detection; thus, this combination is chosen as the sensor construction.

### 4.3. The Influence of Coil Spacing on Detection

**Figure 16:**
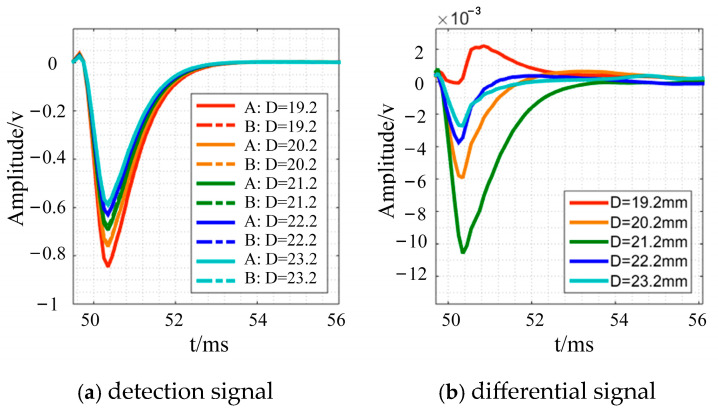
Experimental results under different coil spacing.

The relative distance between the Tx-Rx sensor’s excitation and receiving coils affects the detection signal, so this section compares the detection signal and differential signal variation trend under different coil spacings using the small coil as excitation and the large coil as reception. Figure 16 shows the findings. Figure 16 shows that when D grows, the detected signal diminishes, and the differential signal’s negative semi-axis peak value increases and subsequently falls. Due to D growth, the primary magnetic field formed by the excitation coil directly coupled to the detecting coil has less energy. If the detecting coil can enhance the perception of the specimen’s secondary magnetic field while restricting the primary magnetic field, the detection signal effect improves. Experimental findings suggest that the coil is most sensitive to material thickness change at 21.2 mm, which is ideal for rotating bar defect detection; hence, this is the final sensor spacing.

### 4.4. The Influence of the Relative Position of Coil and Defect on Detection

**Figure 17:**
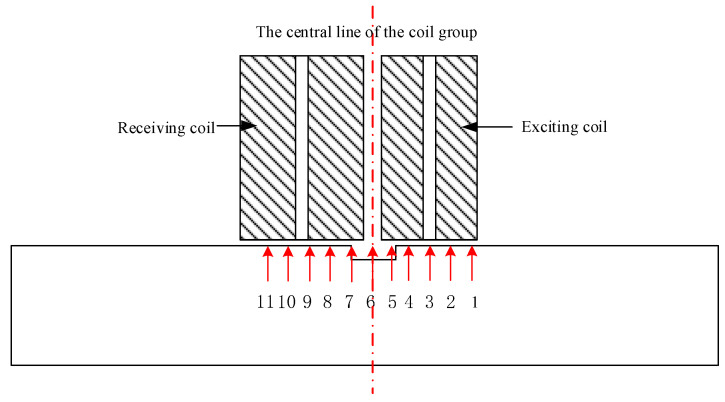
Diagram of sensor position.

**Figure 18:**
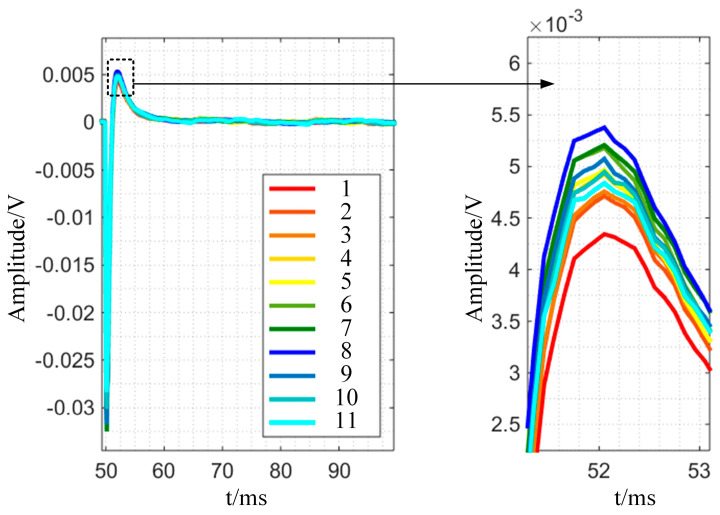
Experimental results of differential signals at different positions.

This section tests the best structural sensor-based detection method on a 20 mm diameter bar with a flat hole defect (diameter 5 mm and depth 2 mm). As shown in Figure 17, move the coil group’s center line from the left to the right side of the defect and place 11 position points on the scan route with a 2 mm gap between them (1, 2, …, 11). As the coil group advances, Figure 18 shows the differential signal amplitude increasing initially and then decreasing at each point. When the coil center line is at position 8, the differential voltage amplitude is highest, and the coil group is most vulnerable to defect-induced eddy current disturbance. In the experiment, the excitation coil covers a greater defect area, which is the best detection technique for the specified coil group.

## 5. Pulsed Eddy Current Testing System and Result Analysis

### 5.1. Experimental System

**Figure 19:**
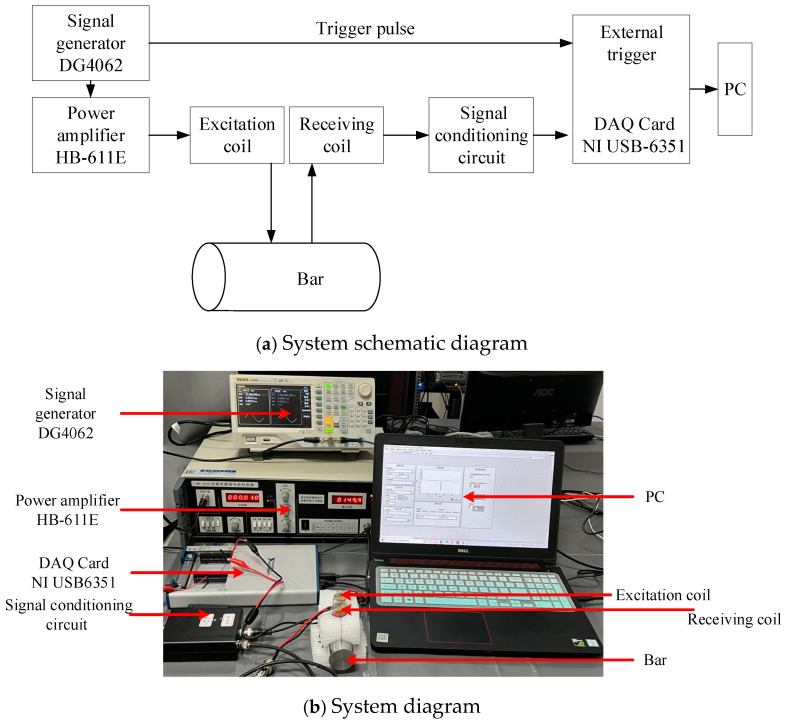
Pulsed eddy current test platform.

Figure 19a illustrates a schematic diagram of a pulsed eddy current testing system utilizing a Tx-Rx sensor. The experimental platform basically comprises a signal generator, a current source, a signal conditioning circuit, a data acquisition system, a Tx-Rx sensor, and a bar. The signal generator is utilized to produce a square wave signal, establish a consistent square wave current via the current source, transfer it to the excitation coil, and stimulate the eddy current field within the specimen. The receiving coil transforms the eddy current field into a voltage signal, which is subsequently amplified and filtered by the signal conditioning circuit, captured by the acquisition card, and eventually displayed and saved by the LabVIEW 2018 application on the computer. The experimental setup utilizes the DG4062 signal generator, the HB-611E power amplifier, and the NI USB-6351 data acquisition device, as seen in Figure 19b. The ten-times average value of each test result is used as a follow-up discussion signal to reduce the interference brought by the experiment.

**Figure 20:**
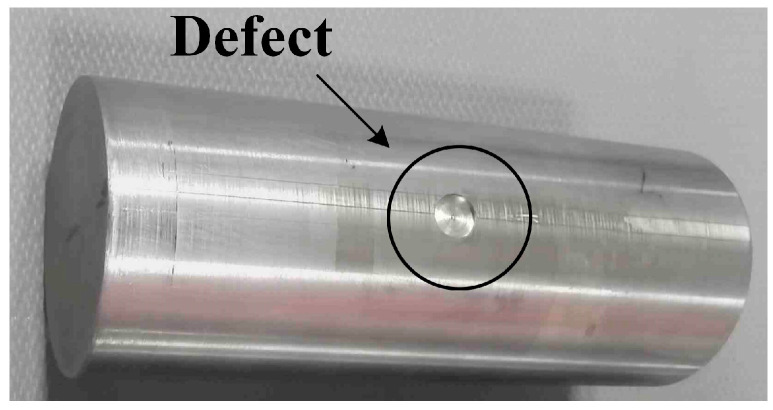
Surface flat hole defect with a depth of 2 mm.

The core of the surface defect is the localized material thinning in certain regions of the bar. Consequently, the bar’s surface is modeled with a diameter of 5 mm and depths of 2 mm, 4 mm, 5 mm, 6 mm, and 8 mm, respectively. The specimen is depicted in Figure 20.

### 5.2. Experimental Results

**Figure 21:**
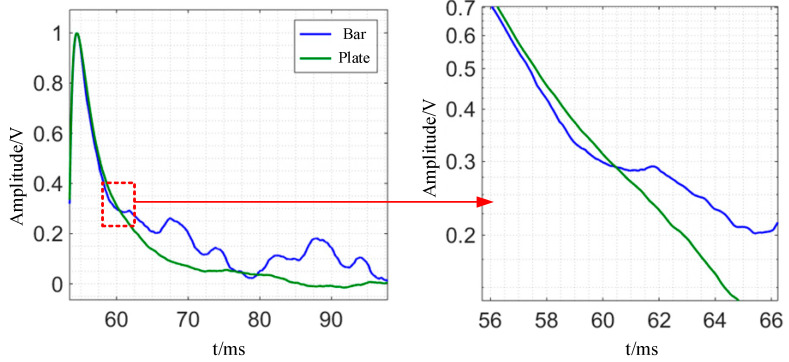
Experimental normalization results of time-domain detection signal of induced voltage of bar and plate.

Experiments are carried out on a steel plate with a thickness of 20 mm and a steel bar with a diameter of 40 mm, respectively, using the experimental platform. The experimental detection signals are normalized in order to compare the changing trend of the two signals more intuitively, and the findings are given in Figure 20. As shown in Figure 21, the detection voltage signal of bar and plate members will attenuate with time, but at different rates: before t = 60.5 ms, the induced voltage signal of plate members is greater than that of bar members; at t = 60.5 ms, the voltage signals of the two shapes are nearly equal; after t = 60.5 ms, the plate member’s induced voltage attenuation is quicker than that of the bar member. Consistent with the simulation analysis results, the eddy current will be reflected at the upper boundary due to the restriction of the circumference boundary of the bar because the effective volume of the bar in space is more concentrated, and the surface area in the electromagnetic field is larger. Because the plate’s horizontal border is far away, the eddy current will be reflected after spreading to the lower surface, causing a shift in the attenuation process.

**Figure 22:**
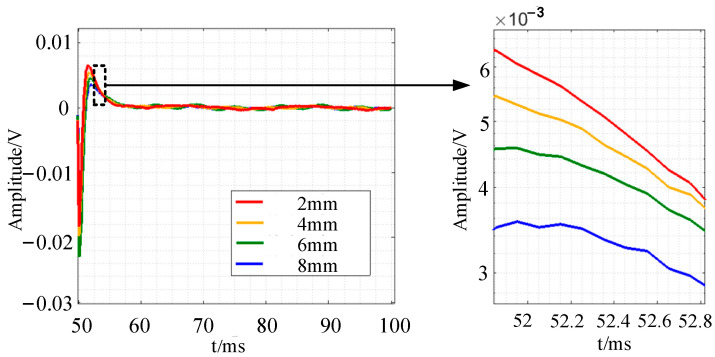
Experimental results of pulsed eddy current testing of defective bars with different depths.

The bar with surface defects of varying depths is further examined in the experiment, and the detection signal of the non-defect region is used as the reference signal, as shown in Figure 22. Figure 22 shows that the signal decays with a normal distribution, especially when the attenuation is steady before and after t = 52 ms. The attenuation stability point extracted from the experimental signal is the reference, the signal slope before and after the point is the characteristic quantity, and the fitting curve of the attenuation slope–defect depth relationship is evaluated in this paper. The slope may be assessed as a characteristic parameter that exclusively pertains to defect thickness information and is unaffected by lift-off. To compute the attenuation slope, five time periods before and after this time point are chosen at random: 51.85 ms, 52.15 ms, 52.35 ms, 52.55 ms, and 52.75 ms. The values are −0.002582, −0.001736, −0.001098, and −0.0005922, respectively. The connection Equation (1) may be obtained by establishing the link between attenuation slope and defect depth:

(1) $$K_{dif}=-2.13\times 10^{-5}{h_{dif}}^{2}+5.43\times 10^{-4}h_{dif}-3.58\times 10^{-3}$$

where *K_dif_*—attenuation slope and *h_dif_*—defect depth.

**Figure 23:**
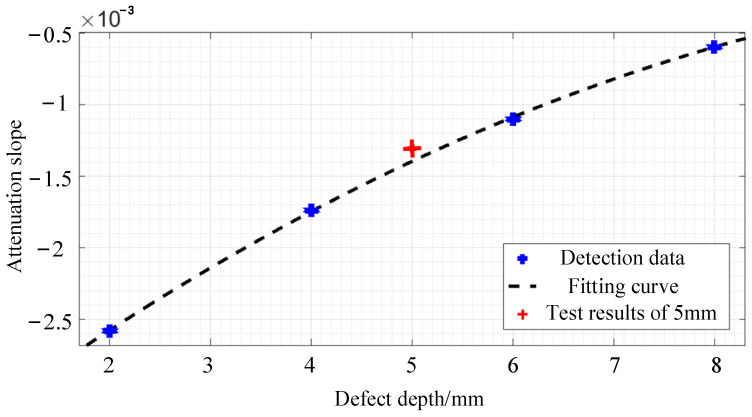
The fitting curve of the relationship between differential signal and attenuation slope of different depth defects.

To assess the dependability of the established functional connection, specimens with a defect depth of 5 mm are chosen for testing, and the corresponding differential signal attenuation slope is entered into Equation (1) for verification, as shown in Figure 23. Refer to Equation (1) to calculate the relative error based on the method, and the results are shown in Table 4. The relative error of the defect depth assessment technique based on attenuation slope is less than 5%, as shown in Table 4, and it has high detection accuracy. The experimental findings demonstrate that by utilizing the fitting curve, the pulsed eddy current testing system can detect surface defects on the bar and estimate the magnitude of the defects.

## 6. Conclusions and Discussion

A Tx-Rx sensor has been created to identify surface defects on small-sized bars, utilizing pulsed eddy current detection technology for this purpose. A 3D model of pulsed eddy current detection is developed with COMSOL 5.6 software. The current density distribution within the plate and bar is analyzed at different frequencies, and the correlation between the excitation coil’s position on the bar and the eddy current disruption around the defect is examined. The characteristics of the pulsed eddy current detection signal are then analyzed. The simulation results indicate that when the excitation coil’s projection on the specimen intersects with the defect, the defect significantly influences the eddy current field on the specimen’s surface, offering guidance for coil positioning.

Additionally, a correlation between differential signal attenuation and defect depth may be established, offering a benchmark for quantitative defect diagnosis. A pulsed eddy current experimental device has been established to detect surface flaws in specimens. The attenuation law of signal detection in plates and bars is analyzed, and the signal variation law under varying depths of surface defects is explored. The formula linking attenuation slope to defect depth for bars is established. The experimental results indicate that the relative inaccuracy of this relation in identifying surface defects on bar specimens is under 5%. The experimental results indicate that the proposed pulsed eddy current detection system is capable of detecting surface defects in bar specimens.

In this paper, the detection effect of the sensor is studied from the optimal coil dimensions, coil separation, and elevation height. However, there are still many interference factors in the actual detection. Therefore, more information on the calibration of the Tx-Rx sensor needs to be studied in future research.

This paper examines the methodology for determining defect size when the defect type is known. However, in practical detection scenarios, the defect type is often unidentified. Therefore, it is essential to investigate defect identification techniques, including those for uniform wall thickness thinning defects and blind holes at the bottom, as well as methods for concurrently ascertaining the depth and radius of flat-bottom blind holes, to enhance the precision of defect evaluation.

## Figures and Tables

**Table 1:** Material properties of models.

Name	Relative Permeability	Electrical Conductivity/(S/m)	Relative Dielectric Constant
45 steel	300	5 × 10^6^	1
Air	1	1	1

**Table 2:** Coil model parameter.

Parameter	Inner Diameter/mm	Outer Diameter/mm	Height/mm	Number of Turns	Wire Diameter/mm
Exciting coil	2	16	30	3800	0.2
Receiving coil	2	22	30	5000	0.2

**Table 3:** Coil parameter.

Sensor	Outer Diameter	Inner Diameter	Height	Number of Turns	Wire Diameter
Small size coil	16 mm	2 mm	30 mm	3800	0.2 mm
Large size coil	22 mm	2 mm	30 mm	5000	0.2 mm

**Table 4:** Comparison of actual defect depth and calculated defect depth results.

Actual Defect Depth	Attenuation Slope	Calculated Defect Depth	Relative Error
5 mm	−0.0013	5.26 mm	4.94%

## Data Availability

Data are contained within the article.
